# Supplementary material for: Identification of photoperiod-induced specific miRNAs in the adrenal glands of Sunite sheep (Ovis aries)
Source: Front Vet Sci. 2022 Jul 22;9:888207. doi: 10.3389/fvets.2022.888207 (PMC9354845; doi:10.3389/fvets.2022.888207)
Supplement: Supplementary file 1 [file Data_Sheet_1.ZIP › Supplementary file/miRDeep2 file.zip!/miRDeep2 file/Know_Structure/SPLP42A3/oar-mir-329b.pdf]

miRBase precursor : oar-mir-329b
Total read count : 1983
oar-miR-329b-5p read count : 27
oar-miR-329b-3p read count : 1949
remaining reads : 7

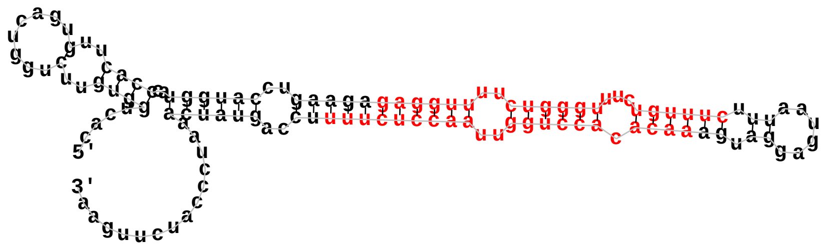

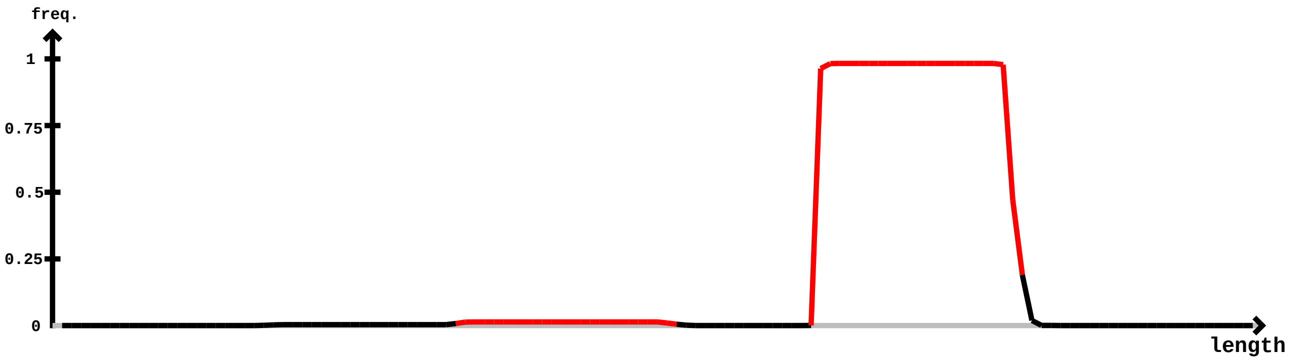


```
                                                                                                                   oar-miR-329b-3p
                                            oar-miR-329b-5p
5'- cacugguguucuggucaguguucacccaugguaccugaagagagguuuucuggguuucuguuucuuuaaugaggaugaaacacaccugguuaaccucuuuuccaguaucaaaucccaucuugaa   -3'   exp
    ....((((..(........)..))))..((((((..((((((((((..((((((...((((((.((......)).)))))).))))))..))))))))))..))))))................    reads   mm
    ....................uucacccaugguaccugaag....................................................................................    1       0
    ....................uucacccaugguaccugaaga...................................................................................    1       0
    .....................ucacccaugguaccugaaga...................................................................................    3       0
    ......................cacccaugguaccugaag....................................................................................    1       0
    ......................cacccaugguaccugaaga...................................................................................    1       0
    ........................................agagguuuucuggguuucuguu..............................................................    5       0
    ........................................agagguuuucuggguuucuguuu.............................................................    4       0
    ........................................agagguuuucuggguuucuguuuc............................................................    1       0
    .........................................gagguuuucuggguuucuguu..............................................................    3       0
    .........................................gagguuuucuggguuucuguuu.............................................................    5       0
    .........................................gagguuuucuggguuucuguuuc............................................................    6       0
    .........................................gagguuuucuggguuucuguuucu...........................................................    2       0
    ..........................................agguuuucuggguuucuguuucu...........................................................    1       0
    .............................................................................aaacacaccugguuaaccucu..........................    1       0
    ..............................................................................aacacaccugguuaaccuc...........................    9       0
    ..............................................................................aacacaccugguuaaccucu..........................    1001    0
    ..............................................................................aacacaccugguuaaccucuu.........................    551     0
    ..............................................................................aacacaccugguuaaccucuuu........................    332     0
    ..............................................................................aacacaccugguuaaccucuuuu.......................    17      0
    ...............................................................................acacaccugguuaaccucu..........................    4       0
    ...............................................................................acacaccugguuaaccucuu.........................    6       0
    ...............................................................................acacaccugguuaaccucuuu........................    7       0
    ...............................................................................acacaccugguuaaccucuuuu.......................    20      0
    ................................................................................cacaccugguuaaccucuuuucc.....................    1       0
```
